# Admission Route and In-Hospital Mortality Among Trauma Patients Admitted to a Regional Trauma Center in South Korea: A Retrospective Cohort Study

**DOI:** 10.3390/jcm15207822

**Published:** 2026-10-10

**Authors:** Hagi Jung, Iljae Wang, Wonwoong Tae, Seokran Yeom, Gibyung Lee, Sungwook Park

**Affiliations:** Department of Emergency Medicine, Pusan National University Hospital, 179, Gudeok-ro, Seo-Gu, Busan 49241, Republic of Korea; jcvhagi@gmail.com (H.J.); jrmr9933@gmail.com (I.W.); aasdf2007@naver.com (W.T.); seokrany@gmail.com (S.Y.)

**Keywords:** trauma, interhospital transfer, trauma center, mortality, emergency medical services, trauma system

## Abstract

**Background:** Studies comparing interhospital transfer with direct admission have reported inconsistent associations with mortality, and Korean evidence is limited. We evaluated admission-route associations with in-hospital mortality among patients admitted to a South Korean regional trauma center and explored mortality-associated factors after transfer. **Methods:** This retrospective receiving-center cohort included trauma admissions during 2022–2024. Patients arriving in cardiac arrest who died without return of spontaneous circulation were excluded. Logistic regression estimated adjusted associations; additional analyses varied adjustment sets and cohort restrictions, and transferred-subgroup analyses were exploratory. **Results:** Among 2464 patients, 1504 arrived directly and 960 were transferred; mortality was 18.6% vs. 12.0%. Transfer was associated with lower adjusted mortality odds (adjusted odds ratio [aOR], 0.54; 95% confidence interval [CI], 0.39–0.75) within this selected receiving-center cohort. Omitting arrival physiology and injury-to-center time yielded an aOR of 0.42 (95% CI, 0.32–0.55). Age and arrival Glasgow Coma Scale (GCS) scores were associated with mortality among transferred patients (aOR, 2.03 [95% CI, 1.68–2.46] for age 70 vs. 60 years and 0.75 [0.71–0.79] per 1-point GCS increase). **Conclusions:** In this receiving-center cohort, transfer was associated with lower in-hospital mortality. Among transferred patients, older age and lower arrival GCS scores were exploratory prognostic markers. These associations should not be interpreted as causal, because transferred patients had to survive until arrival, prehospital severity was unmeasured, and arrival measures may reflect prior care.

## 1. Introduction

Injury is a leading cause of death and disability; in South Korea, external causes, including both unintentional and intentional injuries, accounted for 29,088 deaths (8.0% of all deaths) in 2025 [1]. Trauma systems are organized around the principle that seriously injured patients should reach definitive care without unnecessary delay. The “golden hour” has long been used to express this urgency, although the evidence for a fixed time threshold is limited and the time-sensitivity of care differs across injury patterns [2]. Evidence on the setting of care is more consistent: treatment at a trauma center has been associated with lower mortality among seriously injured patients than treatment at non-trauma hospitals [3], and regional triage guidelines aim to direct patients with major injuries to appropriately resourced facilities [4]. Therefore, ensuring that seriously injured patients reach definitive care through an appropriate pathway is critically important.

South Korea began building a national network of regional trauma centers in 2012, and 17 centers had been designated nationwide by 2017 [5]. National panel-review surveys have documented a decline in the preventable trauma death rate, which was estimated at 13.9% in 2021 [6]. These gains have not eliminated the problem of access. Some injured patients receive initial care at a non-trauma hospital before referral to a regional trauma center. Work on critical care transport and retrieval has emphasized that geography and the organization of transfer services shape access to definitive care [7].

Two admission pathways therefore coexist. Direct transport from the scene avoids an intermediate hospital stop and may shorten the time to operative, endovascular, or intensive care, but it can involve a longer initial journey before any hospital-based assessment [8]. Initial presentation to a nearby hospital permits earlier evaluation and resuscitation, but adds referral and interfacility transport before definitive treatment. Studies comparing these pathways have reached conflicting conclusions. Several found similar mortality in transferred and directly admitted patients [9,10,11], others reported higher mortality after transfer [12,13], and transfer from lower-level to higher-level centers has been associated with lower mortality than continued care at the initial center [14]. Systematic reviews have attributed this heterogeneity partly to differences in study design, comparison groups, and patient populations [15,16]. In particular, cohorts assembled at receiving centers include only patients who survived long enough to be transferred, so the composition of the transferred group reflects both referral decisions and survival before arrival.

The interpretation of these studies also depends on the prehospital and referral systems in which they were conducted. US and Canadian frameworks describe the competencies and scope of practice of prehospital personnel [17,18], while Korean emergency medical technicians work within nationally defined practice limits and field-care guidance [19,20]. Differences in these frameworks and in service organization may limit the direct applicability of findings from other countries to Korea. An earlier study at our institution, covering 2015–2016 during an earlier phase of regional trauma-system development, found numerically lower mortality among transferred than among directly admitted patients with severe injuries, but the difference was not statistically significant [8]. Contemporary data on outcomes by admission route within the Korean trauma system are therefore needed.

In this study, we examined the association between admission route—direct admission from the scene versus interhospital transfer—and in-hospital mortality among trauma patients admitted to a regional trauma center. As a secondary, exploratory objective, we described factors associated with in-hospital mortality within the transferred group. The study describes a selected receiving-center cohort and does not compare direct transport and interhospital transfer as competing strategies from the time of injury.

## 2. Materials and Methods

### 2.1. Study Design and Setting

This retrospective cohort study included trauma patients admitted between 1 January 2022 and 31 December 2024 to the regional trauma center of Pusan National University Hospital, a 1400-bed university-affiliated tertiary hospital in Busan, South Korea. The center was designated in 2013 and opened in 2015 [5], and it treats more than 1000 trauma patients annually (10-year average, 2016–2025), approximately 600 of whom have severe injuries. Emergency surgery, transarterial embolization (TAE), neurosurgical intervention, and intensive care are available 24 h a day. The center serves Busan and Gyeongsangnam-do, where 2369 of the 2464 included patients (96.1%) were injured, including 1501 of 1504 directly admitted patients (99.8%) and 868 of 960 transferred patients (90.4%) (Supplementary Figure S1). Patients injured in other regions, mainly Gyeongsangbuk-do, the Jeolla provinces, and Ulsan, are occasionally accepted when definitive care is not available in their own region; these accounted for 69 transferred patients (7.2%) and 2 directly admitted patients (0.1%). The injury location was unknown for 24 patients. Scene transport is provided by the fire-service-based 119 emergency medical services, whose ambulances are staffed by level 1 or level 2 emergency medical technicians or nurses. When a patient meets the severe trauma criteria of the National Fire Agency standard guidelines for field emergency care, which are based on physiological findings, anatomical findings, and mechanism of injury [20], the EMS crew contacts the trauma center hotline, which is carried 24 h a day, 365 days a year, by an emergency medicine specialist. The hotline physician decides on acceptance on the basis of the red criteria of the 2021 US National Guideline for the Field Triage of Injured Patients [21] and the availability of the center’s medical staff; patients who do not meet these criteria may also be accepted, depending on center capacity, when no other hospital is able to receive them. The two sets of criteria differ mainly in the role of injury mechanism: under the National Fire Agency guidelines, a high-risk mechanism alone qualifies a patient as having severe trauma, whereas the 2021 US guideline classifies mechanism of injury among the yellow (moderate-risk) criteria, so a high-risk mechanism alone was not a basis for acceptance. Interhospital transfers follow the same process: the referring physician contacts the trauma center hotline, and acceptance is decided after review of the patient’s condition. Among the 960 transferred patients, the referring facility, classified according to the Korean Medical Service Act, was a tertiary hospital (a designated general hospital providing highly specialized care) for 156 patients (16.3%), a general hospital (≥100 inpatient beds) for 687 (71.6%), a hospital (≥30 inpatient beds) for 112 (11.7%), and a clinic (primarily outpatient facility) for 3 (0.3%); the facility type was unknown for 2 patients. Thus, both admission routes were determined through hotline communication between the trauma center and the EMS crew or referring physician. The Institutional Review Board of Pusan National University Hospital approved the study (No. 2601-002-158) and waived the need for individual consent for this retrospective review of medical records.

### 2.2. Study Population

All trauma patients who arrived at the center during the study period were screened, and those admitted for treatment of traumatic injuries formed the primary cohort. We excluded patients who arrived in cardiac arrest and died in the emergency department without return of spontaneous circulation (ROSC), either after cardiopulmonary resuscitation (CPR) or without CPR because of injuries incompatible with life or prolonged arrest; patients who achieved ROSC and were admitted were included. Patients were classified by admission route. The direct admission group comprised patients transported from the injury scene by the 119 emergency medical services (EMS), and the transfer group comprised patients who had first received care at another hospital. Because patients who died before reaching the center or who were never transferred could not be identified from our records, the cohort was limited to patients who reached and were admitted to the center. No patient was excluded from the final primary analysis because of missing values in the coded model variables (Supplementary Table S4). No formal sample size calculation was performed; all eligible consecutive admissions during the study period were included.

### 2.3. Data Collection

Data were obtained from the institutional trauma registry maintained in the hospital information system. On arrival, emergency medical technicians stationed at the trauma center entered the information available at presentation, including injury time, chief complaint, level of consciousness, vital signs, injury mechanism, and injury location; trauma registry coordinators subsequently entered data from admission to discharge. These data are transmitted to the Korea Trauma Data Bank (KTDB) as they are entered, so the institutional registry corresponds to the center’s KTDB records. After approval by the Institutional Review Board, the data were extracted from the hospital information system by the hospital’s information technology department. Demographic variables were age and sex. Injury mechanisms were classified as traffic accidents, falls from height (descent to a lower level), ground-level falls (falls on the same level), struck by object, cutting or piercing injuries, and other mechanisms. Injury time was obtained from the 119 EMS run sheet for directly admitted patients and from the referral document for transferred patients; when the referral document did not specify the time, the referring hospital was contacted by telephone. On the EMS run sheet, injury time is recorded for every transport, using information from the patient or family members when available. Injury-to-center time was calculated from the recorded timestamps. In some patients who could not communicate and had no witness or family member aware of the circumstances, the recorded time may have been an estimate.

Systolic blood pressure (SBP) and Glasgow Coma Scale (GCS) scores were the first values recorded on arrival, before any treatment at the trauma center; in transferred patients, they were therefore obtained after referring-hospital care and interhospital transport. SBP was categorized as measurable ≥90 mmHg, measurable <90 mmHg, or unmeasurable despite an attempted measurement. The GCS total was calculated from the recorded eye, verbal, and motor responses. For intubated patients whose verbal response was recorded as T (60 direct admissions and 140 transfers), the analytical GCS total was calculated as the recorded eye and motor scores plus 1, consistent with previous studies that assigned a verbal score of 1 to intubated patients [22]. This approach was chosen over treating the verbal component as missing because the handling of untestable verbal responses remains inconsistent across studies [22]. For patients who had received sedatives or neuromuscular blocking agents before arrival, the GCS measured on arrival was used as recorded, without adjustment. These are trauma-center arrival measures, not measurements of untreated physiology before route selection.

Anatomical injury severity was described using the Injury Severity Score (ISS) and the Abbreviated Injury Scale (AIS), with AIS ≥ 3 injuries identified by body region. Surgery or TAE, intensive care unit (ICU) admission, and length of hospital stay were recorded as post-admission management and outcome variables and were compared descriptively, without interpreting differences as effects of admission route.

### 2.4. Outcome Measures

The primary outcome was in-hospital mortality, defined as death during the index hospitalization. The secondary analysis examined factors associated with in-hospital mortality among transferred patients. In the all-arrival sensitivity analysis, emergency-department deaths without ROSC were also counted as deaths.

### 2.5. Statistical Analysis

Continuous variables were summarized as medians with interquartile ranges (IQRs) and compared using the Mann–Whitney U test. Categorical variables were summarized as counts and percentages and compared using Pearson’s chi-square test, or Fisher’s exact test for sparse cells (facial and external AIS ≥ 3 injuries). Unmeasurable SBP values were excluded from numerical SBP summaries and treated as a separate category in categorical comparisons.

The association between admission route and in-hospital mortality was estimated using multivariable logistic regression, with transfer as the admission-route indicator. The primary model included age, sex, injury mechanism, SBP category, GCS total, injury-to-center time, and AIS ≥ 3 injuries of the head and neck, chest, abdomen and pelvic contents, and extremities and pelvic girdle. These covariates were retained irrespective of their individual statistical significance. ISS was not entered together with the regional AIS indicators because both represent anatomical injury severity. Associations were reported as adjusted odds ratios (aORs) with 95% confidence intervals (CIs). Arrival SBP, GCS, and injury-to-center time may reflect prior treatment, intubation, sedation, deterioration, transport, and referral urgency; they were not treated as pre-route baseline confounders. Accordingly, the primary aOR describes a conditional association in the admitted cohort, not an effect of transfer. A model omitting these three variables was used as an alternative descriptive analysis, which also cannot control unmeasured prehospital severity or eliminate selection bias.

Within the transferred subgroup, an exploratory multivariable model was constructed from candidate predictors with univariable *p* < 0.10 or clinical relevance. The final model included age, injury mechanism, GCS, injury-to-center time, and AIS ≥ 3 injuries of the head and neck and of the abdomen and pelvic contents (Section 3.5); an extended model additionally included SBP category (Supplementary Table S7). Surgery or TAE and ICU admission were not included as covariates in either model because they were post-admission management variables.

Functional form was assessed using a Box–Tidwell approach [23], by simultaneously adding age × ln(age), GCS × ln(GCS), and time × ln(time) to models containing their corresponding linear terms and the other covariates (Supplementary Table S5). All three variables were positive, and natural logarithms were applied without adding a constant. Age was modeled using age and age × ln(age) in both main models, whereas GCS and injury-to-center time were retained as linear terms following these diagnostics. Nonlinear age terms, and the SBP terms in the measurable-SBP sensitivity analysis, were reported as paired log-odds coefficients with standard errors and interpreted jointly. The joint association of the two age terms with mortality was tested with a likelihood-ratio test comparing models with and without both terms (2 df). For clinical interpretation, we also estimated the adjusted odds ratio for age 70 versus 60 years, holding the other covariates constant. Equivalent reparameterization of the paired age terms allowed this contrast and its Wald 95% confidence interval to be obtained directly in MedCalc, without changing the fitted model. This contrast does not imply a constant odds ratio for every 10-year age increase. Injury-to-center time was expressed per 60 min.

Multicollinearity was assessed using ordinary design-matrix variance inflation factors (VIFs), calculated from auxiliary linear regressions using the same analytic samples and predictor columns as the logistic models, with categorical variables represented by indicator variables (Supplementary Table S6). Model degrees of freedom counted slope parameters and excluded the intercept. Discrimination was assessed using the area under the receiver operating characteristic curve (AUC), and calibration using the Hosmer–Lemeshow test and plots of observed versus predicted mortality across risk groups (Supplementary Figures S2 and S3). Model performance was evaluated in the development samples only, without resampling or external validation.

Sensitivity analyses of the primary model (Supplementary Table S1) replaced the AIS indicators with ISS; restricted the cohort to patients with ISS > 15; omitted arrival SBP, GCS, and injury-to-center time, which may be influenced by admission route; modeled SBP continuously using SBP and SBP × ln(SBP) among patients with measurable SBP; or excluded patients whose verbal GCS response was recorded as T. Early-death analyses excluded patients who died at this center within 24 or 48 h of injury, calculated as death time minus injury time. All hospital survivors were retained, and arrival after the respective threshold was not itself an exclusion criterion. A separate all-arrival model added the 334 emergency-department deaths without ROSC and included transfer, linear age, sex, and injury mechanism; injury severity and physiological measures were not included because comparable measurements were unavailable for the additional patients. Early-death exclusions and the all-arrival analysis cannot recover patients who died before transfer, were not transferred, or remained at another hospital, and do not resolve survivor-selection or immortal-time bias. The all-arrival model is particularly limited by differential capture of early deaths between admission routes.

All tests were two-sided, and *p* < 0.05 was considered statistically significant. All statistical analyses were performed using MedCalc version 23.7.2 (MedCalc Software Ltd., Ostend, Belgium).

## 3. Results

### 3.1. Patient Selection

**Figure 1:**
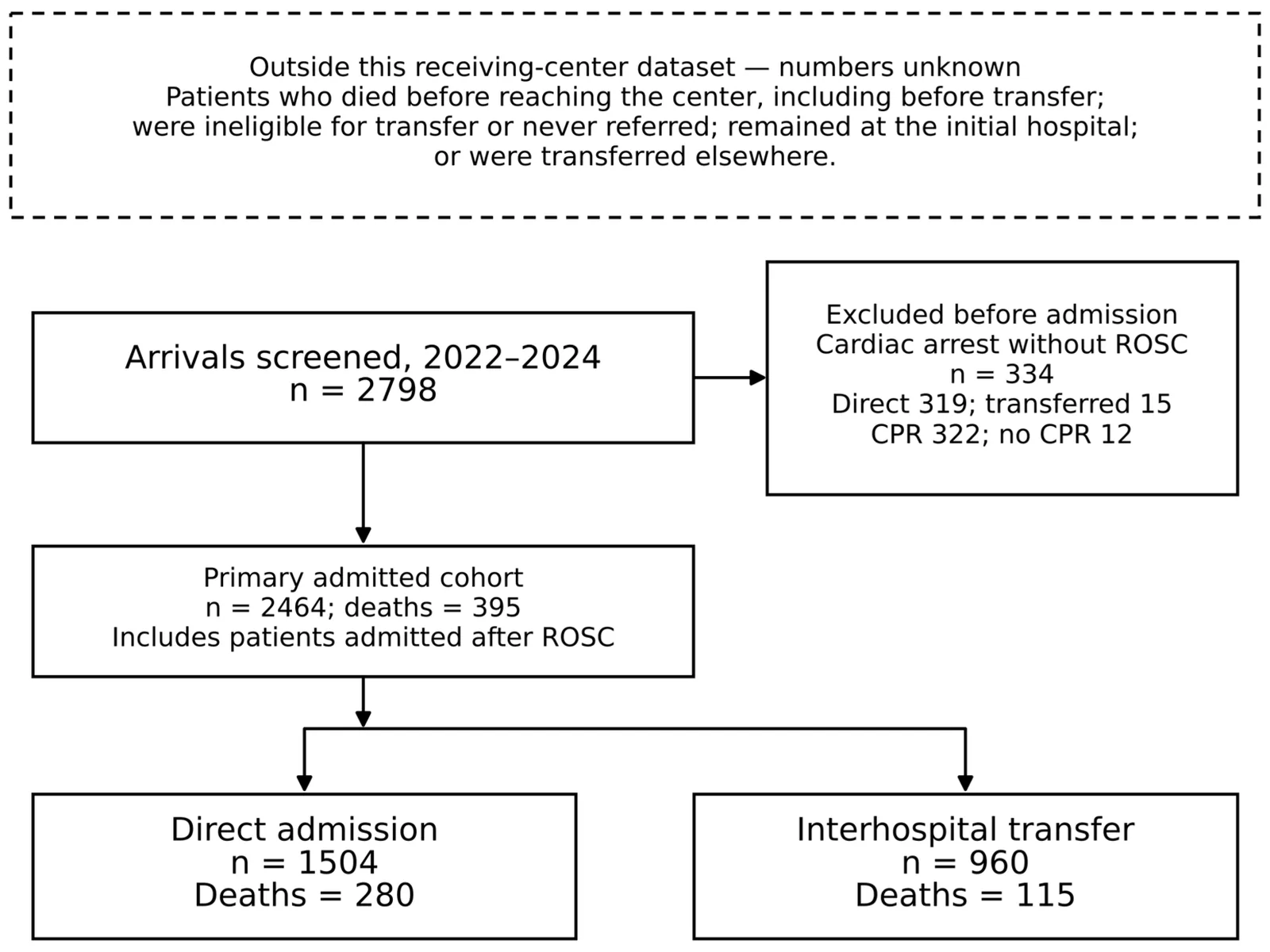
Observed cohort and limits of database coverage. The dashed panel describes patients outside this receiving-center dataset; their numbers are unknown and they are not counted among the 334 observed exclusions. The primary cohort excludes arrivals in cardiac arrest who died without ROSC before admission. Of the 334 excluded patients, 322 underwent CPR and 12 did not because of injuries incompatible with life or prolonged arrest. Patients who achieved ROSC and were admitted were included. ROSC, return of spontaneous circulation; CPR, cardiopulmonary resuscitation.

Among 2798 screened arrivals, 334 arrived in cardiac arrest and died in the emergency department without ROSC (322 after CPR and 12 in whom CPR was not attempted because of injuries incompatible with life or prolonged arrest): 319 had arrived directly and 15 had been transferred. These patients were not admitted. The primary cohort therefore contained 2464 admissions, comprising 1504 direct admissions (280 deaths) and 960 transfers (115 deaths). Patients admitted after ROSC remained in this cohort (Figure 1).

### 3.2. Characteristics According to Admission Route

Transferred patients were older than direct admissions (median 62.0 vs. 57.0 years) and had longer injury-to-center intervals (254.0 vs. 51.0 min; both *p* < 0.001). GCS was higher in transferred patients (median 15.0 vs. 14.0; *p* < 0.001). Unmeasurable SBP occurred in 28 transfers (2.9%) and 130 direct admissions (8.6%). Among patients with measurable SBP, both groups had a median of 110 mmHg, with different distributions (*p* = 0.035). Median ISS did not differ significantly (*p* = 0.241). In-hospital mortality was 12.0% in transfers and 18.6% in direct admissions (Table 1).

### 3.3. Admission Route and Mortality in the Admitted Cohort

In the primary model, transfer was associated with lower adjusted odds of death (aOR 0.54, 95% CI 0.39–0.75; *p* < 0.001; Table 2) within this selected receiving-center cohort. Relative to measurable SBP ≥ 90 mmHg, SBP < 90 mmHg and unmeasurable SBP had aORs of 1.67 (95% CI 1.15–2.41) and 5.26 (3.25–8.53), respectively. Higher GCS was associated with lower odds of death (aOR per point 0.74, 95% CI 0.71–0.76; *p* < 0.001). Age was modeled using age and age × ln(age); the adjusted odds ratio for 70 versus 60 years was 2.03 (95% CI 1.81–2.28; *p* < 0.001). In the alternative descriptive model omitting arrival SBP, GCS, and injury-to-center time, the transfer aOR was 0.42 (95% CI 0.32–0.55; Supplementary Table S3). Neither estimate represents a causal effect of transfer.

The model included 395 deaths and 17 slope degrees of freedom. Apparent AUC was 0.913 (95% CI 0.901–0.924); Hosmer–Lemeshow χ^2^ was 3.393 (*p* = 0.907). Grouped apparent calibration is shown in Supplementary Figure S2. VIFs were 342.714 and 344.038 for the two age terms and 1.035–1.251 for the other predictor columns (Supplementary Table S6).

### 3.4. Comparison Between Survivors and Non-Survivors Among Transferred Patients

Among 960 transferred patients, 115 died. Non-survivors were older and had lower GCS and higher ISS than survivors (Table 3). Median GCS was 3.0 (IQR 3.0–12.0) in non-survivors and 15.0 (14.0–15.0) in survivors (*p* < 0.001). SBP was unmeasurable in 11 non-survivors (9.6%) and 17 survivors (2.0%). Among those with measurable SBP, medians were 120 and 110 mmHg, respectively (*p* = 0.159). Injury-to-center time was shorter in non-survivors (207.0 vs. 260.0 min; *p* < 0.001).

### 3.5. Mortality Associations Within the Transferred Subgroup

The exploratory transferred-subgroup model included 960 patients, 115 deaths, and 11 slope degrees of freedom (Table 4). Age was modeled nonlinearly, and higher GCS was associated with lower odds of death (aOR per point 0.75, 95% CI 0.71–0.79; *p* < 0.001). The adjusted odds ratio for age 70 versus 60 years was 2.03 (95% CI 1.68–2.46; *p* < 0.001). The remaining individual coefficients did not meet the *p* < 0.05 threshold. Apparent AUC was 0.899 (95% CI 0.878–0.917), and Hosmer–Lemeshow χ^2^ was 9.566 (*p* = 0.297). These in-sample assessments are descriptive and do not establish predictive validity. Supplementary Figure S3 shows grouped apparent calibration. VIFs were 277.197 and 278.110 for the two age terms and 1.021–1.227 for the other predictor columns (Supplementary Table S6).

Adding SBP category to the same subgroup specification yielded a GCS aOR of 0.76 (95% CI 0.72–0.80; *p* < 0.001). Compared with measurable SBP ≥ 90 mmHg, unmeasurable SBP was associated with higher mortality odds (aOR 3.86, 95% CI 1.35–11.07; *p* = 0.012), whereas the estimate for measured SBP <90 mmHg was less precise (aOR 1.66, 95% CI 0.89–3.10; *p* = 0.109). The extended model had 13 slope degrees of freedom and a −2 log likelihood of 440.497, compared with 447.923 for the subgroup model (Table 4) (likelihood-ratio χ^2^ = 7.426, 2 df; *p* = 0.024). Full results are provided in Supplementary Table S7.

### 3.6. Sensitivity Analyses

Additional analyses described associations under different cohort definitions and adjustment sets (Supplementary Table S1); their estimates are not directly comparable causal effects. Replacing AIS indicators with ISS gave an aOR of 0.58 (95% CI 0.42–0.80). Excluding deaths within 24 or 48 h of injury gave aORs of 0.61 (0.43–0.86) and 0.62 (0.43–0.90), respectively. The 24-h exclusion removed 103 direct-admission deaths (6.8% of 1504) and 23 transfer deaths (2.4% of 960); the 48-h exclusion removed 146 (9.7%) and 42 (4.4%), respectively. Excluding 200 verbal-T cases gave an aOR of 0.60 (0.41–0.90). Including non-ROSC emergency-department deaths in a separately specified limited model gave an aOR of 0.26 (0.21–0.33). Omitting arrival SBP, GCS, and injury-to-center time gave an aOR of 0.42 (95% CI 0.32–0.55); restricting the cohort to patients with ISS > 15 gave 0.50 (0.36–0.72); and modeling SBP continuously among patients with measurable SBP gave 0.59 (0.42–0.84). The early-death exclusions and all-arrival analysis do not resolve survivor-selection or immortal-time bias. The all-arrival estimate is highly limited because the added early deaths were predominantly direct admissions (319 direct vs. 15 transferred), while deaths before transfer remained unobserved. Cohort counts, adjustment sets, and transfer estimates are summarized in Supplementary Table S1. Full coefficients for the ISS alternative adjustment and the model omitting arrival physiology and time are reported in Supplementary Tables S2 and S3, respectively.

## 4. Discussion

In this retrospective cohort of 2464 trauma patients admitted to a regional trauma center in South Korea, interhospital transfer was associated with lower in-hospital mortality after adjustment for measured covariates, despite a longer interval from injury to center arrival. The alternative descriptive model omitting arrival physiology and time also estimated an association, but neither model resolves selection before arrival or unmeasured prehospital severity. Within the transferred subgroup, age and GCS were associated with mortality. However, because these findings describe patients who reached and were admitted to the receiving center, they do not estimate the effect of assigning direct transport or interhospital transfer at the time of injury.

Previous studies have reached differing conclusions about transfer and mortality. Hamada et al. found no significant mortality difference between direct transport and secondary transfer in a French trauma system [9]. Hsiao et al. likewise reported that transfer after stabilization was not independently associated with mortality in their severe-trauma population [10], while Rozenberg et al. reported comparable mortality after motor-vehicle trauma despite differences in injury severity [11]. In contrast, Tiruneh et al. reported higher mortality among transferred patients than among direct admissions to Level I and II centers [12], and Boschini et al. found an adverse association with indirect transfer in a sub-Saharan African setting [13]. Our finding therefore differs from most previous direct-versus-transfer comparisons, which reported similar or higher mortality after transfer. These differences may reflect access to definitive care, referral practices, patient selection, and the care available before arrival. They also caution against interpreting admission route as an exposure with the same meaning in every trauma system.

The comparator is particularly important when relating our results to those of Waalwijk et al. [14]. Their study examined severely injured patients initially taken to lower-level centers and compared subsequent transfer with remaining at a lower-level center. It therefore addresses access to higher-level care after initial hospital presentation, rather than the direct-versus-transfer comparison among patients admitted to one receiving center.

The present findings also extend an earlier report from our institution by Yang et al. [8]. In that smaller cohort of 634 severely injured patients from 2015–2016, mortality was numerically lower among transfers than among direct admissions, although the difference was not statistically significant. The current cohort covers a later period and includes a broader range of injury severity. The recurring direction of association merits further investigation of local referral and treatment pathways, but the larger sample and statistical significance do not themselves establish a transport benefit. Changes in case mix, referral practice, and trauma-system organization may affect comparisons between periods. Both cohorts came from the same institution and may share the same selection mechanisms; this is not independent external validation.

The observed association may reflect selection into the receiving-center cohort, differences in care before arrival, or both; this study cannot establish their relative contributions. Referring-hospital care could include airway support, hemorrhage control, resuscitation, and diagnostic assessment that help stabilize a patient before onward transport. Most transferred patients (87.8%) had initially presented to tertiary or general hospitals, although the trauma capabilities of individual referring facilities were not recorded. Conversely, patients who survive the initial injury, remain eligible for transfer, and complete interhospital transport are a selected subset of those initially presenting elsewhere. Patients who die before transfer, are considered too unstable, or are never referred are absent from the transferred group. Selection of this kind has been identified as a major methodological concern in receiving-center studies of interhospital transfer [15,16]. In addition, because patients with a high-risk mechanism but without red criteria were not routinely accepted directly, some physiologically stable patients may have reached the center only after initial care elsewhere, contributing to the more favorable arrival physiology of the transferred group. Either process, or their combination, could contribute to the observed association. We did not record the relevant interventions or serial physiological measurements and therefore cannot determine their relative contributions.

The arrival measurements illustrate this ambiguity. Transferred patients had higher GCS scores and a smaller proportion of unmeasurable SBP, but both groups had the same median measurable SBP of 110 mmHg. These values were recorded before treatment at our center, yet after referring-hospital care and transport for transferred patients. They cannot be treated as direct evidence that prior stabilization improved survival. Similar median ISS values, together with differences in the distribution of regional injuries, also do not establish comparability at the scene or before referral. Anatomical scores cannot fully represent physiological instability, evolving injury, or the clinical judgments underlying transfer decisions.

Injury-to-center time showed a similar pattern. Transferred patients arrived a median of 254 min after injury, compared with 51 min for direct admissions; most transferred patients (59.2%) were injured in Gyeongsangnam-do, outside the city in which the center is located, which partly explains their longer intervals. Nevertheless, elapsed time was not independently associated with mortality in the primary model (aOR per hour 0.994, 95% CI 0.984–1.004). Within the transferred group, non-survivors arrived earlier than survivors (median 207 vs. 260 min). This pattern is consistent with severity-driven prioritization, in which patients who appear more critically ill are referred and transported more rapidly, rather than with a protective effect of longer intervals. Similarly, a large prospective North American cohort found no association between prehospital time intervals and mortality among injured patients with physiological abnormality [24]. These findings do not establish that delay is safe or distinguish severity-driven referral from other selection processes.

The Korean trauma-system setting remains relevant to interpreting these observations. In this cohort, direct admissions arrived through the 119 service, whereas transferred patients had first been evaluated at another hospital. The potential role of that hospital encounter depends on available clinical capabilities, the patient’s condition, and access to definitive treatment. However, the present data do not show that hospital-based stabilization compensated for a longer journey or that any specific EMS arrangement explains the mortality difference. Context-specific evidence is therefore needed before findings from this center can inform regional transport policy.

The sensitivity analyses help describe how the association changes with specific analytical choices (Supplementary Table S1). Replacing regional AIS indicators with ISS provided an alternative representation of injury burden, and the ISS > 15 analysis examined patients with major trauma. Omitting arrival SBP, GCS, and elapsed time avoided adjustment for those particular downstream variables, but did not create a fully controlled comparison of pre-exposure characteristics. The two models yielded different conditional associations (aOR 0.42 without these variables vs. 0.54 with them); this comparison does not identify a causal mechanism or quantify bias. The 24- and 48-h analyses excluded deaths at this center within the specified interval from injury; they were not formal landmark analyses and further conditioned the cohort on survival. Including the 334 emergency-department deaths without ROSC broadened the receiving-center population, but required limited adjustment because comparable severity and physiological measurements were unavailable. The all-arrival estimate is especially difficult to interpret because early deaths at this center were predominantly captured among direct admissions (319 vs. 15), whereas deaths before transfer were not captured. Neither early-death exclusions nor the all-arrival model can recover patients who died before transfer, were ineligible for transfer, remained at the initial hospital, or were transferred elsewhere. Estimates below 1 therefore do not establish robustness to immortal-time, survivor-selection, or referral bias.

The transferred-subgroup findings provide a separate, exploratory, and hypothesis-generating description of mortality risk after arrival. Lower GCS was associated with mortality in both the subgroup model (Table 4) and the model additionally including SBP categories; unmeasurable SBP was also associated with mortality in the extended model (Supplementary Table S7). Age was modeled nonlinearly and should be interpreted through its combined terms rather than a constant per-year odds ratio. The earlier institutional study identified pretransfer intubation as a prognostic marker [8], whereas the present analysis describes neurological and circulatory status at receiving-center arrival. These observations concern related aspects of clinical severity, but are not interchangeable: intubation, sedation, and prior resuscitation can influence the measured arrival state.

Across the adjusted models, lower arrival GCS and unmeasurable SBP were consistently associated with in-hospital mortality, both in the full cohort (aOR per GCS point 0.74; unmeasurable SBP aOR 5.26) and among transferred patients (0.75 and 3.86, respectively). Because these measurements followed referring-hospital care in transferred patients, they are best interpreted as prognostic markers at arrival rather than as baseline severity or as mechanisms explaining the transfer association.

The distinction between prognosis and transport benefit is important for clinical interpretation. Sewalt et al. found that predicted benefit from Level I trauma-center care varied with patient characteristics, including head and thoracic injury and signs of shock [25]. Kim et al. reported age-stratified associations between trauma-center transport and survival in Korea, including an association in older patients with traumatic brain injury [26]. These studies address trauma-center access in populations and comparisons different from ours. Our subgroup analysis identifies associations among patients who have already been transferred; it does not compare the outcomes those same patients would have experienced under direct transport. These associations describe prognostic markers at receiving-center arrival; their clinical predictive utility was not validated. These hypothesis-generating findings can help frame future research on high-risk transferred patients, but do not support prioritizing older or low-GCS patients for a particular route on the basis of this study alone, or delaying definitive care to obtain stabilization at another hospital.

This study has several limitations. The retrospective, single-center design restricts generalizability to centers with different referral populations and prehospital services. To facilitate comparison with other systems, we reported route-specific cohort and death counts for each analysis. More fundamentally, eligibility follows the transport pathway: transferred patients must survive initial care, acceptance, and transport before entering the admitted cohort. Scene physiology, EMS triage decisions, travel distance, referring-hospital interventions and capabilities, transfer indications, and reasons for delayed or absent transfer were not available for systematic comparison. These factors may influence both route and mortality, and selection and unmeasured confounding may explain part or all of the observed association. The early-death exclusions and all-arrival model vary the observed cohort but cannot test whether these biases account for the association, because patients who died before transfer or were never transferred remain absent. Propensity-score or inverse probability weighting methods were not used because they rely on the same measured covariates and cannot address the absence of patients who were never transferred. Adjustment for arrival physiology and elapsed time can also introduce overadjustment or collider bias because these variables may be consequences of route and preceding care.

Measurement limitations require additional caution. Unmeasurable SBP was represented as its own category rather than an arbitrary numerical pressure, but the category does not capture the degree or duration of circulatory compromise. The same 90-mmHg cutoff was used across ages and is not an age-specific pediatric hypotension definition. For intubated patients with an untestable verbal response, the analytical GCS total used the recorded eye and motor responses plus 1. An untestable verbal response limits interpretation of the total score [22]. Excluding verbal-T cases changes the case mix and does not validate the convention or eliminate effects of sedation and neuromuscular blockade. Recorded injury times may be imprecise when the patient could not communicate and no witness or family member could establish the timing. The analytic dataset did not identify how injury time had been estimated in these cases.

Finally, the models were developed and evaluated in the same data. Functional-form checks examined specified transformations and cannot exclude other nonlinear relationships. The high VIFs for age and age × ln(age) reflect strong dependence between the jointly specified age terms; their coefficients should not be interpreted separately. Other predictor-column VIFs were low. The transferred-subgroup models had approximately 10.5 and 8.8 events per slope parameter (115 deaths for 11 and 13 parameters), which may increase the risk of overfitting. The subgroup model was selected partly using univariable results, and the reported confidence intervals and *p*-values do not account for the model-selection process. Apparent AUC and calibration may therefore be optimistic and should not be taken as external validation.

A study of transport effectiveness would require a common time zero before route selection, such as first EMS contact, and linked prehospital, referring-hospital, transfer, receiving-center, and mortality data that include patients never transferred. Such a design could examine whether particular patient groups benefit from a transport strategy while distinguishing prognosis from treatment benefit. The analytic dataset, extracted from our hospital information system, contained no patient-level information linking our records with those of referring hospitals, so the condition of transferred patients before transfer could not be determined. In Korea, the national trauma registry (KTDB) records scene vital signs and level of consciousness from 119 EMS records, as well as vital signs and GCS on arrival at each hospital, including referring hospitals [27]. However, although hospitals submit identifiable records to the KTDB, personal identifiers are removed before data are released to individual centers to protect privacy, so such linkage is not possible through the registry either. Linkage of prehospital, referring-hospital, and trauma-center data, including patients who were never transferred, would be required to evaluate transport strategies [15,16].

## 5. Conclusions

Among patients who reached and were admitted to this regional trauma center, interhospital transfer was associated with lower in-hospital mortality. Among transferred patients, older age and lower arrival GCS were associated with higher mortality; these were exploratory prognostic markers, not evidence for route-specific treatment recommendations. These associations should not be interpreted as causal, because of selection before cohort entry, unmeasured prehospital severity, and adjustment for arrival measures influenced by prior care. Evaluating transport strategies will require linked prehospital, referring-hospital, and receiving-center data that include patients never transferred and use a common time zero before route selection.

## Figures and Tables

**Table 1:** Characteristics, post-admission management, and outcomes by admission route.

Variable	Direct Admission (*n* = 1504)	Transferred (*n* = 960)	*p*-Value
Age, years	57.0 (40.0–68.0)	62.0 (47.0–72.0)	<0.001
Age group, years			<0.001
0–17	61 (4.1)	58 (6.0)	
18–64	929 (61.8)	496 (51.7)	
65+	514 (34.2)	406 (42.3)	
Sex			0.282
Male	1089 (72.4)	714 (74.4)	
Female	415 (27.6)	246 (25.6)	
Mechanism of injury			<0.001
Traffic accidents	693 (46.1)	436 (45.4)	
Fall from height	441 (29.3)	241 (25.1)	
Ground-level fall	67 (4.5)	150 (15.6)	
Struck by object	87 (5.8)	80 (8.3)	
Cutting or piercing	162 (10.8)	32 (3.3)	
Others	54 (3.6)	21 (2.2)	
At trauma center arrival			
SBP, mmHg (measurable only)	110.0 (90.0–130.0)	110.0 (90.0–135.0)	0.035
SBP category			<0.001
≥90 mmHg	1051 (69.9)	768 (80.0)	
<90 mmHg	323 (21.5)	164 (17.1)	
Unmeasurable	130 (8.6)	28 (2.9)	
GCS total score	14.0 (7.0–15.0)	15.0 (12.0–15.0)	<0.001
GCS category			<0.001
3–8	429 (28.5)	199 (20.7)	
9–12	147 (9.8)	43 (4.5)	
13–15	928 (61.7)	718 (74.8)	
Injury-to-center time, minutes	51.0 (38.0–70.0)	254.0 (180.3–389.0)	<0.001
ISS	17.0 (10.0–26.0)	17.0 (11.0–25.0)	0.241
ISS > 15	971 (64.6)	641 (66.8)	0.260
Injury region with AIS ≥ 3			
Head and neck	330 (21.9)	252 (26.3)	0.014
Face	0 (0.0)	1 (0.1)	0.390
Chest	350 (23.3)	270 (28.1)	0.007
Abdomen and pelvic contents	185 (12.3)	200 (20.8)	<0.001
Extremities and pelvic girdle	125 (8.3)	50 (5.2)	0.004
External	1 (0.1)	1 (0.1)	1.000
Post-admission management and outcomes			
Surgery or TAE	491 (32.6)	383 (39.9)	<0.001
ICU admission	1199 (79.7)	841 (87.6)	<0.001
In-hospital length of stay, days	16.0 (4.0–34.0)	18.0 (9.0–31.5)	0.001
In-hospital mortality	280 (18.6)	115 (12.0)	<0.001

Values are *n* (%) or median (IQR). SBP medians use 1374 direct admissions and 932 transfers with measurable values; SBP-category percentages use the full group denominators. For intubated patients with a verbal GCS component coded as T, the verbal component was assigned a value of 1 for calculation of the analytical GCS total. Facial and external AIS indicators were compared using Fisher exact tests. AIS, Abbreviated Injury Scale; ISS, Injury Severity Score; SBP, systolic blood pressure; GCS, Glasgow Coma Scale; TAE, transarterial embolization; ICU, intensive care unit.

**Table 2:** Multivariable associations with in-hospital mortality in the admitted cohort.

Variable	aOR (95% CI) or B (SE)	*p*-Value
Age, B (SE)	−0.373 (0.0691)	<0.001
Age × ln(age), B (SE)	0.0858 (0.0141)	<0.001
Age 70 vs. 60 years, derived contrast	2.03 (1.81–2.28)	<0.001
Female vs. male	0.80 (0.57–1.10)	0.171
Mechanism of injury (reference: traffic accidents)		
Fall from height	0.95 (0.68–1.33)	0.750
Ground-level fall	1.55 (0.94–2.53)	0.083
Struck by object	0.80 (0.39–1.64)	0.549
Cutting or piercing	1.07 (0.52–2.19)	0.861
Other mechanism	0.61 (0.25–1.49)	0.281
Transfer vs. direct admission	0.54 (0.39–0.75)	<0.001
SBP < 90 mmHg	1.67 (1.15–2.41)	0.007
SBP unmeasurable	5.26 (3.25–8.53)	<0.001
GCS total score, per point	0.74 (0.71–0.76)	<0.001
Injury-to-center time, per hour	0.994 (0.984–1.004)	0.208
Head and neck AIS ≥ 3	1.63 (1.20–2.21)	0.002
Chest AIS ≥ 3	1.16 (0.83–1.63)	0.383
Abdomen and pelvic contents AIS ≥ 3	0.99 (0.62–1.59)	0.968
Extremities and pelvic girdle AIS ≥ 3	1.07 (0.58–1.95)	0.834

*N* = 2464; deaths = 395; direct 1504/280 deaths; transferred 960/115 deaths; slope df = 17. Reference categories are male sex, traffic accident, direct admission, SBP ≥ 90 mmHg, and regional AIS < 3. ORs are mutually adjusted. Age and age × ln(age) must be interpreted jointly (joint likelihood-ratio χ^2^ = 177.8, 2 df, *p* < 0.001); B denotes a log-odds coefficient and SE its standard error. The age contrast (70 vs. 60 years) is derived from an equivalent reparameterization of the same model, not an additional predictor or a constant per-decade association. Arrival SBP and GCS are prognostic measurements after the prehospital pathway. Time is expressed per 60 min. aOR, adjusted odds ratio; CI, confidence interval.

**Table 3:** Characteristics and post-admission management of transferred patients by survival.

Variable	Survivors (*n* = 845)	Non-Survivors (*n* = 115)	*p*-Value
Age, years	60.0 (44.0–70.0)	73.0 (63.0–81.0)	<0.001
Age group, years			<0.001
0–17	56 (6.6)	2 (1.7)	
18–64	465 (55.0)	31 (27.0)	
65+	324 (38.3)	82 (71.3)	
Sex			0.728
Male	630 (74.6)	84 (73.0)	
Female	215 (25.4)	31 (27.0)	
Mechanism of injury			<0.001
Traffic accidents	393 (46.5)	43 (37.4)	
Fall from height	205 (24.3)	36 (31.3)	
Ground-level fall	120 (14.2)	30 (26.1)	
Struck by object	77 (9.1)	3 (2.6)	
Cutting or piercing	31 (3.7)	1 (0.9)	
Others	19 (2.2)	2 (1.7)	
At trauma center arrival			
SBP, mmHg (measurable only)	110.0 (90.0–130.0)	120.0 (90.0–150.0)	0.159
SBP category			<0.001
≥90 mmHg	689 (81.5)	79 (68.7)	
<90 mmHg	139 (16.4)	25 (21.7)	
Unmeasurable	17 (2.0)	11 (9.6)	
GCS total score	15.0 (14.0–15.0)	3.0 (3.0–12.0)	<0.001
GCS category			<0.001
3–8	119 (14.1)	80 (69.6)	
9–12	36 (4.3)	7 (6.1)	
13–15	690 (81.7)	28 (24.3)	
Injury-to-center time, minutes	260.0 (187.0–398.0)	207.0 (151.0–333.0)	<0.001
ISS	17.0 (10.0–25.0)	25.0 (18.3–29.0)	<0.001
ISS > 15	537 (63.6)	104 (90.4)	<0.001
Injury region with AIS ≥ 3			
Head and neck	197 (23.3)	55 (47.8)	<0.001
Face	0 (0.0)	1 (0.9)	0.120
Chest	245 (29.0)	25 (21.7)	0.105
Abdomen and pelvic contents	188 (22.2)	12 (10.4)	0.003
Extremities and pelvic girdle	43 (5.1)	7 (6.1)	0.651
External	1 (0.1)	0 (0.0)	1.000
Post-admission management and outcomes			
Surgery or TAE	326 (38.6)	57 (49.6)	0.024
ICU admission	731 (86.5)	110 (95.7)	0.005

Values are *n* (%) or median (IQR). SBP medians use 828 survivors and 104 non-survivors with measurable values; SBP-category percentages use all 845 survivors and 115 non-survivors. For intubated patients with a verbal GCS component coded as T, the verbal component was assigned a value of 1 for calculation of the analytical GCS total. Facial and external AIS indicators were compared using Fisher exact tests. Abbreviations are as in Table 1.

**Table 4:** Multivariable associations with in-hospital mortality among transferred patients.

Variable	aOR (95% CI) or B (SE)	*p*-Value
Age, B (SE)	−0.310 (0.108)	0.004
Age × ln(age), B (SE)	0.0737 (0.0221)	<0.001
Age 70 vs. 60 years, derived contrast	2.03 (1.68–2.46)	<0.001
Mechanism of injury (reference: traffic accidents)		
Fall from height	1.26 (0.70–2.29)	0.438
Ground-level fall	1.15 (0.59–2.22)	0.680
Struck by object	0.54 (0.14–2.03)	0.362
Cutting or piercing	0.59 (0.06–5.50)	0.644
Other mechanism	0.45 (0.08–2.47)	0.355
GCS total score, per point	0.75 (0.71–0.79)	<0.001
Injury-to-center time, per hour	0.996 (0.985–1.008)	0.512
Head and neck AIS ≥ 3	1.54 (0.91–2.59)	0.105
Abdomen and pelvic contents AIS ≥ 3	1.15 (0.54–2.46)	0.725

*N* = 960; deaths = 115; slope df = 11. All participants were transferred. Reference categories are traffic accidents and regional AIS < 3. ORs are adjusted for the other listed terms; the model in Table 4 does not adjust for SBP category. The extended model including SBP category is reported in Supplementary Table S7. Age and age × ln(age) are interpreted jointly (joint likelihood-ratio χ^2^ = 66.0, 2 df, *p* < 0.001). The age contrast (70 vs. 60 years) is derived from the same fitted model and is not a constant per-decade association. This exploratory model describes prognostic associations at receiving-center arrival and does not identify patients who would benefit from a particular transport strategy. Time is expressed per 60 min. aOR, adjusted odds ratio; CI, confidence interval; B, log-odds coefficient; SE, standard error. Abbreviations are as in Table 1.

## Data Availability

The datasets generated and/or analyzed during the current study are not publicly available due to hospital policy but are available from the corresponding author on reasonable request.

## Supplementary Materials

The following supporting information can be downloaded at: https://www.mdpi.com/article/10.3390/jcm15207822/s1. Figure S1: Geographic context of the regional trauma center and study area; Figure S2: Grouped apparent calibration of the primary model; Figure S3: Grouped apparent calibration of the transferred-subgroup model; Table S1: Admission-route associations across the primary and sensitivity analyses; Table S2: ISS alternative adjustment; Table S3: Arrival physiology and time omitted; Table S4: Availability of coded variables in the admitted analytic cohort; Table S5: Functional-form diagnostics and final model specification; Table S6: Variance inflation factors for the two main model design matrices; Table S7: Extended transferred-subgroup model including SBP categories.
