# Supplementary material for: Admission Route and In-Hospital Mortality Among Trauma Patients Admitted to a Regional Trauma Center in South Korea: A Retrospective Cohort Study
Source: J Clin Med. 2026 Oct 10;15(20):7822. doi: 10.3390/jcm15207822 (PMC13705049; doi:10.3390/jcm15207822)
Supplement: Supplementary file 1 [file jcm-15-07822-s001.zip › jcm-4537577-supplementary.pdf]

Supplementary information

Admission Route and In-Hospital Mortality Among Trauma Patients Admitted to a Regional Trauma Center in South Korea: A Retrospective Cohort Study

Table S1. Admission-route associations across the primary and sensitivity analyses

Analysis / adjustment	All n / deaths	Direct n / deaths	Transfer n / deaths	Transfer aOR (95% CI)	p	df
Primary AIS model (A)	2,464 / 395	1,504 / 280	960 / 115	0.54 (0.39–0.75)	<0.001	17
ISS alternative adjustment (B)	2,464 / 395	1,504 / 280	960 / 115	0.58 (0.42–0.80)	0.001	14
ISS >15 subgroup (A)	1,612 / 365	971 / 261	641 / 104	0.50 (0.36–0.72)	<0.001	17
Exclusion of deaths within 24 hours of injury (A)	2,338 / 269	1,401 / 177	937 / 92	0.61 (0.43–0.86)	0.005	17
Exclusion of deaths within 48 hours of injury (A)	2,276 / 207	1,358 / 134	918 / 73	0.62 (0.43–0.90)	0.012	17
Arrival physiology and time omitted (C)	2,464 / 395	1,504 / 280	960 / 115	0.42 (0.32–0.55)	<0.001	13
Measurable SBP with nonlinear SBP terms (D)	2,306 / 303	1,374 / 199	932 / 104	0.59 (0.42–0.84)	0.003	17
GCS verbal T cases excluded (A)	2,264 / 305	1,444 / 239	820 / 66	0.60 (0.41–0.90)	0.013	17
All arrivals including non-ROSC ED deaths (E)	2,798 / 729	1,823 / 599	975 / 130	0.26 (0.21–0.33)	<0.001	8

All estimates compare transfer with direct admission. These analyses use different cohorts and adjustment sets, do not estimate causal effects, and should not be compared as directly comparable effect sizes. Estimates below 1 do not demonstrate robustness to survivor-selection, immortal-time, or referral bias. The all-arrival analysis includes deaths before admission and uses limited adjustment; early deaths are differentially captured by admission route, while deaths before transfer remain unobserved. The remaining analyses concern the admitted cohort. Early-death exclusions are based on injury-to-death time, not time from center arrival. The 24-hour exclusion removed 103 direct and 23 transferred patients; the 48-hour exclusion removed 146 and 42, respectively.

Adjustment sets: A, age and age × ln(age), sex, injury mechanism, SBP category, GCS total, injury-to-center time in hours, and four AIS ≥3 indicators (head/neck, chest, abdomen/pelvic contents, extremities/pelvic girdle). B, set A with ISS replacing all four AIS indicators. C, set A without SBP, GCS, or injury-to-center time. D, set A with numerical SBP and SBP × ln(SBP) replacing SBP categories, restricted to measurable SBP. E, linear age, sex, and injury mechanism only. Transfer is included in every model. Natural logarithms were used. df counts slope parameters, excluding the intercept.

Early-death exclusions retain all hospital survivors and do not exclude patients solely for arrival after the threshold; they are not formal landmark analyses. The verbal-T exclusion removes 60 direct and 140 transferred patients whose GCS total used E + M + 1. The all-arrival model adds 319 direct and 15 transferred patients who died without ROSC before admission; valid comparable injury-severity and physiological measurements were unavailable for these additional patients. Neither these exclusions nor the all-arrival analysis recovers patients who died before transfer, were ineligible for transfer, remained at the referring hospital, or were transferred elsewhere.

Table S2. ISS alternative adjustment

Variable	aOR (95% CI) or B (SE)	p-value
Age, B (SE)	−0.400 (0.0728)	<0.001
Age × ln(age), B (SE)	0.0915 (0.0149)	<0.001
Female vs male	0.77 (0.55–1.08)	0.130
Fall from height	0.97 (0.70–1.36)	0.880
Ground-level fall	1.65 (1.01–2.71)	0.045
Struck by object	0.94 (0.46–1.92)	0.864
Cutting or piercing	1.54 (0.72–3.31)	0.265
Other mechanism	0.81 (0.34–1.95)	0.635
Transfer vs direct admission	0.58 (0.42–0.80)	0.001
SBP <90 mmHg	1.48 (1.02–2.13)	0.037
SBP unmeasurable	4.64 (2.90–7.43)	<0.001
GCS total score, per point	0.75 (0.72–0.77)	<0.001
Injury-to-center time, per hour	0.995 (0.986–1.004)	0.285
ISS, per point	1.04 (1.03–1.05)	<0.001

N=2,464; deaths=395; slope df=14; full-model −2 log likelihood=1238.962. Direct: 1,504 patients/280 deaths; transferred: 960/115.

ISS replaced all four AIS-region indicators.

Reference categories: male sex, traffic accidents, direct admission, measurable SBP ≥90 mmHg. Each listed OR is adjusted for the other listed terms. B denotes a log-odds coefficient; SE denotes its standard error. Nonlinear term pairs must be interpreted together. Natural logarithms were used. Time effects are per 60 minutes.

Table S3. Arrival physiology and time omitted

Variable	aOR (95% CI) or B (SE)	p-value
Age, B (SE)	−0.239 (0.0558)	<0.001
Age × ln(age), B (SE)	0.0560 (0.0114)	<0.001
Female vs male	0.94 (0.72–1.22)	0.638
Fall from height	0.95 (0.72–1.25)	0.712
Ground-level fall	1.12 (0.75–1.67)	0.593
Struck by object	0.63 (0.36–1.12)	0.117
Cutting or piercing	0.59 (0.34–1.04)	0.067
Other mechanism	0.69 (0.33–1.47)	0.338
Transfer vs direct admission	0.42 (0.32–0.55)	<0.001
Head and neck AIS ≥3	2.64 (2.05–3.39)	<0.001
Chest AIS ≥3	1.25 (0.95–1.63)	0.108
Abdomen and pelvic contents AIS ≥3	0.96 (0.67–1.37)	0.814
Extremities and pelvic girdle AIS ≥3	1.31 (0.83–2.05)	0.243

N=2,464; deaths=395; slope df=13; full-model −2 log likelihood=1888.630. Direct: 1,504 patients/280 deaths; transferred: 960/115.

SBP, GCS, and injury-to-center time were omitted.

Reference categories: male sex, traffic accidents, direct admission, regional AIS <3. Each listed OR is adjusted for the other listed terms. B denotes a log-odds coefficient; SE denotes its standard error. Nonlinear term pairs must be interpreted together. Natural logarithms were used.

Table S4. Availability of coded variables in the admitted analytic cohort

Variable	Available coded records	Unavailable coded records
In-hospital mortality	2,464	0
Admission route	2,464	0
Age	2,464	0
Sex	2,464	0
Injury mechanism	2,464	0
SBP category	2,464	0
GCS analytical total	2,464	0
Injury-to-center time	2,464	0
Head and neck AIS ≥3	2,464	0
Chest AIS ≥3	2,464	0
Abdomen and pelvic contents AIS ≥3	2,464	0
Extremities and pelvic girdle AIS ≥3	2,464	0
ISS	2,464	0

The selected coded analysis variables were available for all 2,464 admitted patients. SBP was unmeasurable despite an attempted measurement in 158 patients (130 direct, 28 transferred) and was represented by a separate category; numerical SBP was available in 2,306 patients. In 200 intubated patients (60 direct, 140 transferred), the verbal GCS component was T and was coded as 1 for the analytical total. For the additional 334 non-ROSC emergency-department deaths, ISS was unassessed; recorded zero values were not interpreted as true ISS scores.

Table S5. Functional-form diagnostics and final model specification

Added term	Primary model p	Transferred subgroup p	Specification used
Age × ln(age)	<0.001	<0.001	Age and age × ln(age) retained
GCS × ln(GCS)	0.149	0.859	Linear GCS retained
Time × ln(time)	0.254	0.156	Linear time in hours retained

Each diagnostic model added all three transformation terms alongside their corresponding linear terms and the other covariates. The primary diagnostic included 2,464 patients/395 deaths (19 slope df); the transferred-subgroup diagnostic included 960 patients/115 deaths (13 slope df). The retained models in Tables 2 and 4 have 17 and 11 slope df, respectively. These p-values concern the transformation terms, not overall tests of the corresponding predictors. Natural logarithms were used. For the clinically interpretable age contrasts in Tables 2 and 4, define $U=(age-60)/10$ and $V=age\times\ln(age)-60\times\ln(60)-U\times[70\times\ln(70)-60\times\ln(60)]$. Replacing age and age×ln(age) with U and V preserves the model space and fitted probabilities. The coefficient of U is the adjusted log-odds contrast for age 70 versus 60 years; exponentiating it and its Wald confidence limits yields the reported odds ratio and 95% CI. The primary model gave 2.0322 (1.8080–2.2842), and the transferred-subgroup model gave 2.0312 (1.6755–2.4625); both $p<0.0001$. This contrast is not a constant effect per decade.

SBP was categorical in the primary model. In the separate measurable-SBP analysis (2,306 patients/303 deaths; 17 slope df), the linear SBP term had $B=-0.263$ ($SE=0.0488$) and SBP × ln(SBP) had $B=0.0455$ ($SE=0.00846$); both $p<0.001$. These terms must be interpreted jointly. Its transfer estimate is reported in Table S1.

Table S6. Variance inflation factors for the two main model design matrices

Predictor column	Primary cohort n=2,464	Transferred subgroup n=960
Age	342.714	277.197
Age × ln(age)	344.038	278.110
Sex	1.051	Not included
Transfer	1.142	Not included
Fall from height	1.178	1.188
Ground-level fall	1.221	1.227
Struck by object	1.087	1.103
Cutting or piercing	1.157	1.053
Other mechanism	1.060	1.037
SBP <90 mmHg	1.081	Not included
SBP unmeasurable	1.201	Not included
GCS total score	1.251	1.112
Injury-to-center time	1.046	1.021
Head and neck AIS ≥3	1.142	1.171
Chest AIS ≥3	1.062	Not included
Abdomen and pelvic contents AIS ≥3	1.095	1.111
Extremities and pelvic girdle AIS ≥3	1.035	Not included

Ordinary design-matrix VIFs were calculated with multiple linear regression using the same samples and predictor columns as the logistic models. Injury mechanism and SBP categories were dummy-coded. The primary and subgroup models used 17 and 11 predictor columns, respectively. High VIFs for age and age × ln(age) reflect dependence between the jointly interpreted age terms. Other predictor-column VIFs ranged from 1.035 to 1.251 and from 1.021 to 1.227, respectively. VIF, variance inflation factor.

Table S7. Extended transferred-subgroup model including SBP categories

Variable	aOR (95% CI) or B (SE)	p-value
Age, B (SE)	−0.290 (0.106)	0.006
Age × ln(age), B (SE)	0.0698 (0.0217)	0.001
Fall from height	1.37 (0.75–2.50)	0.313
Ground-level fall	1.41 (0.71–2.80)	0.325
Struck by object	0.49 (0.12–2.01)	0.323
Cutting or piercing	0.46 (0.04–5.41)	0.536
Other mechanism	0.59 (0.10–3.33)	0.548
GCS total score, per point	0.76 (0.72–0.80)	<0.001
Injury-to-center time, per hour	0.995 (0.985–1.006)	0.394
Head and neck AIS ≥3	1.69 (0.99–2.87)	0.053
Abdomen and pelvic contents AIS ≥3	1.04 (0.48–2.28)	0.914
SBP <90 mmHg	1.66 (0.89–3.10)	0.109
SBP unmeasurable	3.86 (1.35–11.07)	0.012

N=960; deaths=115; survivors=845; all patients were transferred. Slope df=13; full-model −2 log likelihood=440.497. This model retains every predictor in Table 4 and adds two SBP-category indicators; the subgroup model (Table 4) and this extension use the same patients.

Reference categories: traffic accidents, regional AIS <3, and measurable SBP ≥90 mmHg. ORs are adjusted for the other listed terms. Age and age × ln(age) must be interpreted jointly; B denotes a log-odds coefficient and SE its standard error. Natural logarithms were used. Time effects are per 60 minutes.

Relative to Table 4, adding the two SBP indicators reduced −2 log likelihood from 447.923 to 440.497 (likelihood-ratio χ^2=7.426, 2 df; p=0.024, calculated from the displayed likelihoods). Apparent AUC was 0.901 (95% CI 0.880–0.919); Hosmer–Lemeshow χ^2=9.436, 8 df, p=0.307. These are in-sample assessments, not external validation. Figure S3 and the transferred-subgroup VIFs in Table S6 refer to the model in Table 4, not this extension.

Figure S1. Geographic context of the regional trauma center and study area

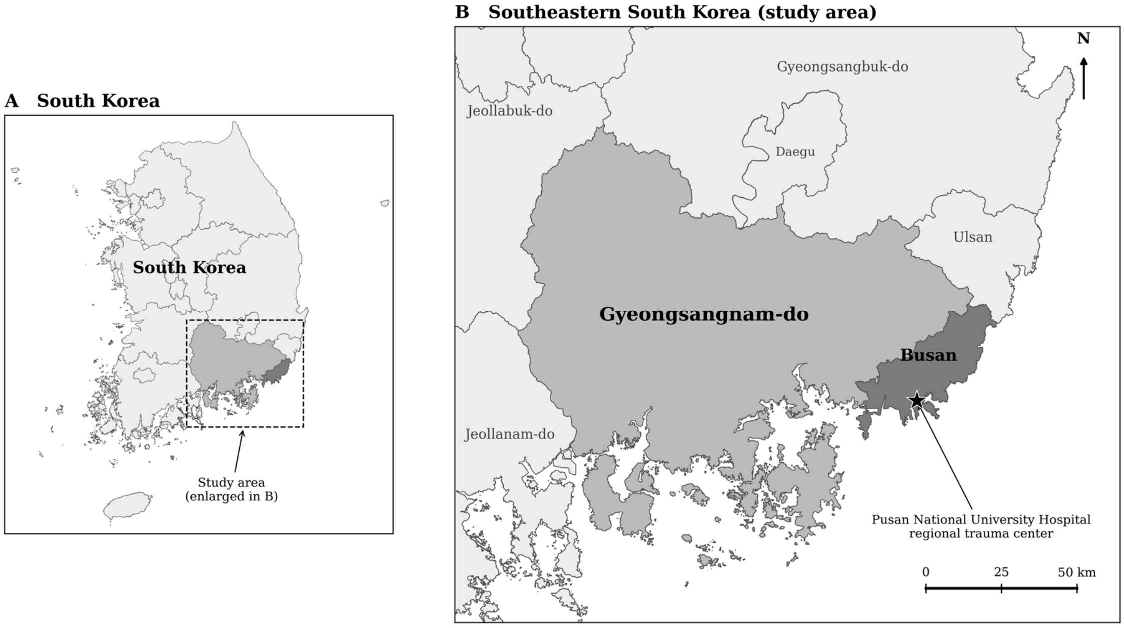

Busan and Gyeongsangnam-do are shown to provide geographic context for the study setting. The star indicates Pusan National University Hospital Regional Trauma Center in Busan. The map is provided for geographic context only and does not represent individual transport routes or measured transport distances. The map was created by the authors in Python (matplotlib) using provincial administrative boundary data from Statistics Korea (2013 boundaries; southkorea-maps open-source repository).

Figure S2. Grouped apparent calibration of the primary model

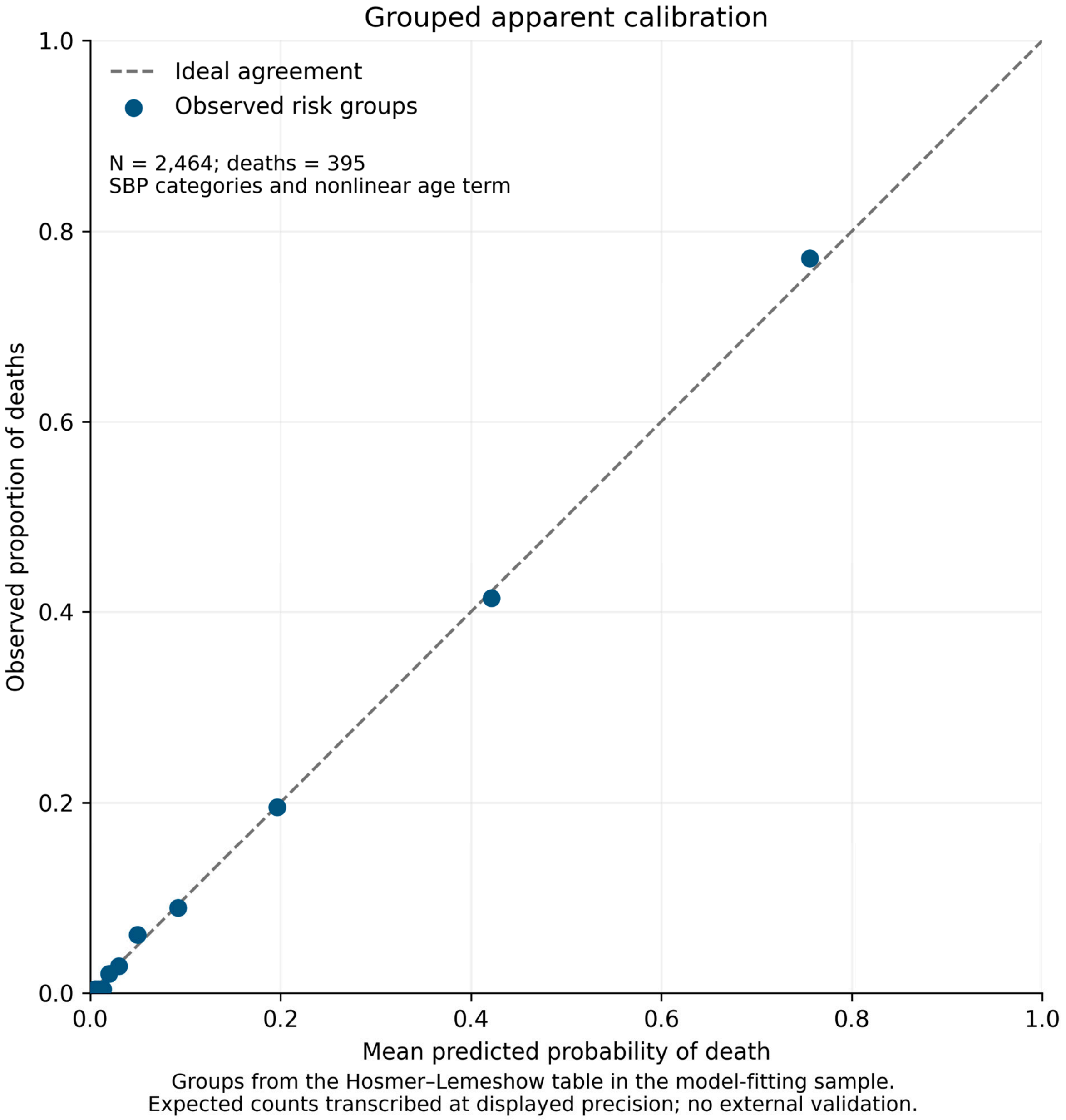

Observed mortality is plotted against mean predicted probability in approximately ten risk groups. The dashed line denotes equality. Predictions and outcomes are from the model-development sample (n=2,464; deaths=395). AUC (95% CI): 0.913 (0.901–0.924); Hosmer–Lemeshow χ^2=3.393; p=0.907. These grouped plots do not represent external or optimism-corrected validation.

Figure S3. Grouped apparent calibration of the transferred-subgroup model

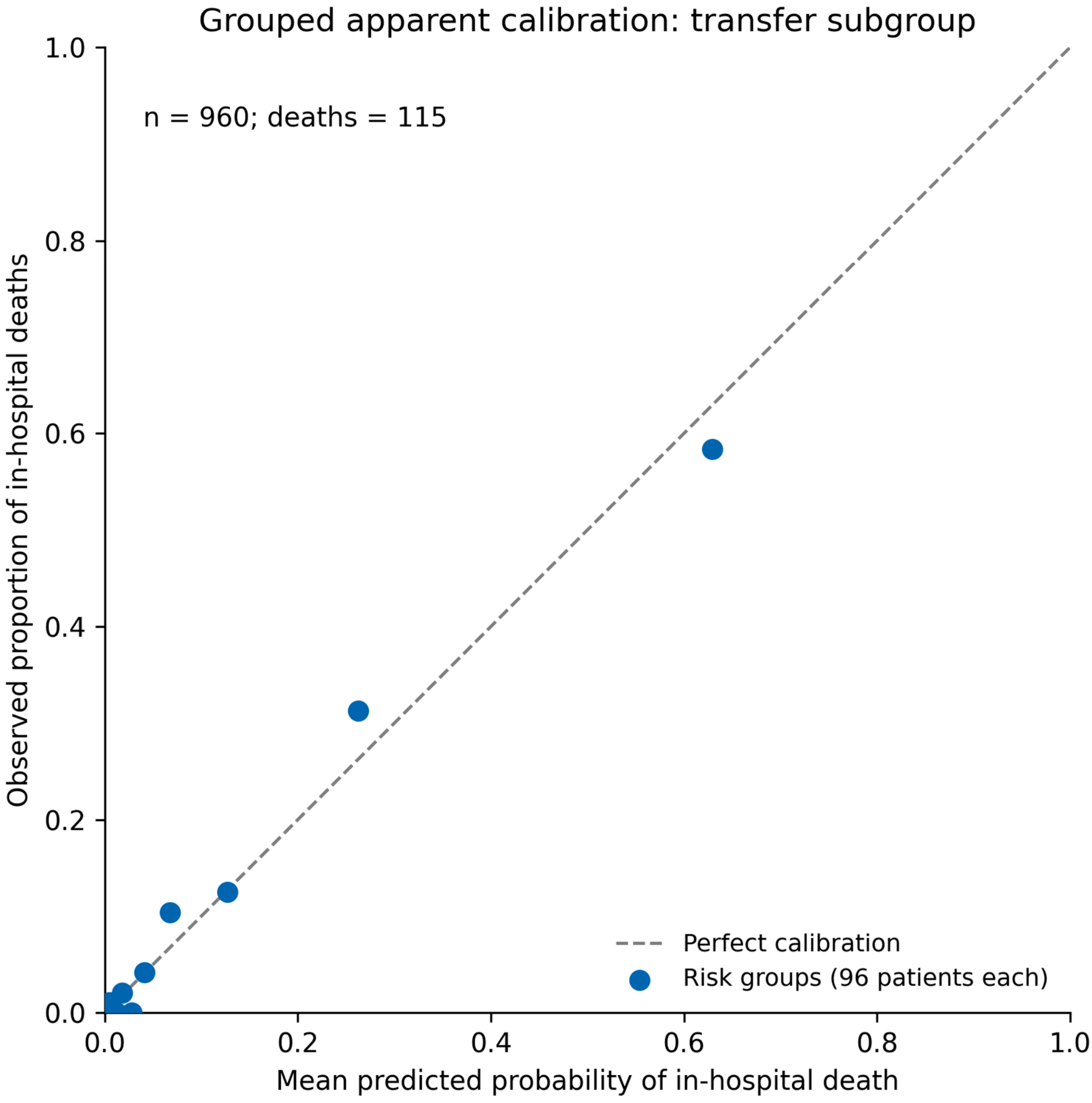

Observed mortality is plotted against mean predicted probability in approximately ten risk groups. The dashed line denotes equality. Predictions and outcomes are from the model-development sample (n=960; deaths=115). AUC (95% CI): 0.899 (0.878–0.917); Hosmer–Lemeshow χ^2=9.566; p=0.297. These grouped plots do not represent external or optimism-corrected validation.
